# Proteo-Transcriptomic Characterization of the Venom from the Endoparasitoid Wasp *Pimpla turionellae* with Aspects on Its Biology and Evolution

**DOI:** 10.3390/toxins11120721

**Published:** 2019-12-10

**Authors:** Rabia Özbek, Natalie Wielsch, Heiko Vogel, Günter Lochnit, Frank Foerster, Andreas Vilcinskas, Björn Marcus von Reumont

**Affiliations:** 1 Project group Bioressources, Animal Venomics, Fraunhofer Institute for Molecular Biology and Applied Ecology, Winchesterstrasse 2, 35392 Giessen, Germany; 2 Research Group Mass Spectrometry/Proteomics, Max Planck Institute for Chemical Ecology, Hans-Knoell-Strasse 8, 07745 Jena, Germany; 3 Department of Entomology, Max Planck Institute for Chemical Ecology, Hans-Knoell-Strasse 8, 07745 Jena, Germany; hvogel@ice.mpg.de; 4 Protein Analytics, Institute of Biochemistry, Justus Liebig University, Friedrichstrasse 24, 35392 Giessen, Germany; guenter.lochnit@biochemie.med.uni-giessen.de; 5 Bioinformatics Core Facility, Bioinformatics and Systems Biology, Justus Liebig University, Heinrich Buff Ring 58, 35394 Giessen, Germany; 6 Institute for Insect Biotechnology, Justus Liebig University, Heinrich Buff Ring 58, 35394 Giessen, Germany; 7 LOEWE Centre for Translational Biodiversity Genomics (LOEWE-TBG), Senckenberganlage 25, 60325 Frankfurt, Germany

**Keywords:** hymenopteran venomics, parasitoid wasps, proteomics, transcriptomics, Pimplin2, ICK, knottins

## Abstract

Within mega-diverse Hymenoptera, non-aculeate parasitic wasps represent 75% of all hymenopteran species. Their ovipositor dual-functionally injects venom and employs eggs into (endoparasitoids) or onto (ectoparasitoids) diverse host species. Few endoparasitoid wasps such as *Pimpla turionellae* paralyze the host and suppress its immune responses, such as encapsulation and melanization, to guarantee their offspring’s survival. Here, the venom and its possible biology and function of *P. turionellae* are characterized in comparison to the few existing proteo-transcriptomic analyses on parasitoid wasp venoms. Multiple transcriptome assembly and custom-tailored search and annotation strategies were applied to identify parasitoid venom proteins. To avoid false-positive hits, only transcripts were finally discussed that survived strict filter settings, including the presence in the proteome and higher expression in the venom gland. *P. turionella* features a venom that is mostly composed of known, typical parasitoid enzymes, cysteine-rich peptides, and other proteins and peptides. Several venom proteins were identified and named, such as pimplin2, 3, and 4. However, the specification of many novel candidates remains difficult, and annotations ambiguous. Interestingly, we do not find pimplin, a paralytic factor in *Pimpla hypochondriaca*, but instead a new cysteine inhibitor knot (ICK) family (pimplin2), which is highly similar to known, neurotoxic asilid1 sequences from robber flies.

## 1. Introduction

Hymenoptera constitutes a mega-diverse insect order that is well known for its vast number of species (~150,000 according to reference [1], in which venom evolved for predation, defense, and communication [2,3,4]. They feature multiple life-style forms as solitary or social pollinators, predators, and parasitoids [4,5,6]. However, studied in more detail since the 1950s especially, are the venom components from a few aculeate species that occur in closer proximity to humans, such as eusocial bees and wasps (Apidae and Vespidae). For example, in aculeate hymenopterans, considered the original ovipositor, as the egg-laying structure was modified as from an apomorph character in this group to a stinger to employ venom exclusively from a connected venom gland system [4,6,7]. Nonetheless, more than 75% of known hymenopteran species are non-aculeate, i.e., are parasitoid wasps (parasitoids) that still utilize the ovipositor in its original function to lay eggs and “weaponize” it in a dual function to inject venom into host species they parasitize [8,9,10,11]. In stark contrast to aculeate venom, which is streamlined for defense to immobilize or to kill their prey [7,12,13,14], venom of parasitoids mainly alters the physiology and behavior of the host to keep it alive while feeding the offspring [15,16,17,18,19]. Despite this interesting biology, only a few parasitoid venom systems were studied in more detail.

Ectoparasitoids that lay eggs outside/on the host normally induce paralysis with their venom to ensure a successful feeding of the larvae; the time scale of the paralysis can vary [10,11,17,20,21,22]. The practical “zombification” of hosts that is induced by some species reflects a climax to ensure the successful development of their larvae. A prominent example is the jewel wasp *Ampulex compressa* that injects venom into the central nervous system of American cockroaches. The sting results in lethargy and hypokinesia accompanied by the suppression of any escape reflex without altering other behavior [19,23]. Proteomics analyses indicate that the neuropeptides tachykinin and corazonin induce these effects [19].

**Figure 1:**
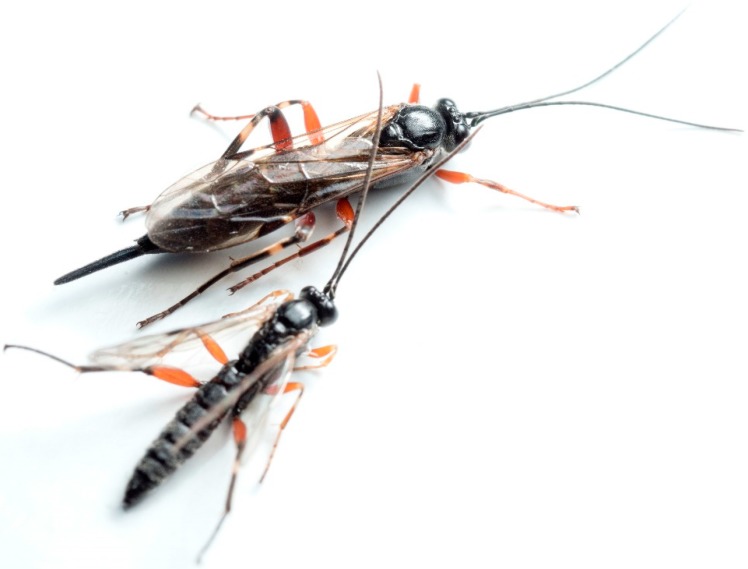
Female and male specimens of *P. turionellae*. The larger female is seen at the top. Males do not show typical female characteristics, such as the prominent ovipositor, and, therefore, also lack the venom system.

In contrast, idiobiont endoparasitoids, such as the herein studied taxon *Pimpla turionellae* (see Figure 1), induce eggs into the host, and their venoms are rather designed to interfere with the host’s immune system and development [17]. Parasitoids evolved diverse strategies to attack specific stages of the hosts and either stop (idiobiont) or allow the host to continue (koinobiont) its development [24]. The specific parasitization of host stages and the ability of parasitoids to manipulate host physiology at the behavioral [23], endocrinal [25], nutritional [26], or immunological level [27] evoked a strong interest in their venom components for pharmaceutical and agrochemical research.

It might be reasoned by the small size and miniaturization of most parasitoids that only for few species are the venom, and its composition analyzed in more depth, despite studies on the general effects of envenomation dating back over four decades [7,17,28]. Details of specific components and single proteins derive mostly from proteome work that started more extensively in the 1980s [7,10,17,29]. Nonetheless, slowly proteo-transcriptomic data analyses that combine proteomics and transcriptomics are becoming well published for selected taxa [17,29], see Supplementary Table S1. A general picture is that the main venom components of many parasitoids are proteases and their inhibitors can involve and impair normal host physiology to guarantee the survival of the parasitoid’s offspring [16,29,30,31]. Other biomolecules complement the venom cocktail, such as small peptides, mid-to high-molecular-weight enzymes, protease inhibitors, recognition/ binding proteins, immune-related proteins, neurotoxin-like peptides and paralytic factors [17,22,32]. The known biological function and identification source of major venom components of endoparasitoids are summarized in Supplementary Table S1.

From an evolutionary and biological perspective, ectoparasitism represents the ancestral form in parasitoid hymenopterans, which evolved the first time in orussid woodwasps, the closest relative to Apocrita [6]. The rapid radiation of parasitoid lineages in the natural group of Parasitoida [6] that comprises of primarily parasitoid wasps is besides miniaturization and a wasp waist (better maneuverability to position the sting), also linked to a diverse venom evolution and adaptation to the ecology and biology of hosts. In endoparasitoids, the paralyzing function of the venom becomes less important because the eggs are laid into the host, and the offspring no longer needs physical protection outside the host, i.e., by paralyzing it. However, by introducing the offspring into the host, parasitoid survival is jeopardized by the exposure to the host’s immune system. A typical immune response to neutralize an intruding factor such as parasitoid progeny normally involves encapsulation by “enveloping” the egg with layers of hemocytes. This reaction is often associated in parallel with melanization, a process in which melanin pigments are deposited on an invading parasite or pathogen [11]. Most known and activity tested venom components of endoparasitoids manipulate the pathways and cascades that are related to these immune responses, such as the inactivation of hemocytes or melanization [17,33]. One of the endoparasitoids in which the venom and its components are better studied is *P. hypochondriaca* [17,34,35,36,37,38,39,40,41,42,43,44], despite the fact that many of its venom proteins remain unstudied, and thus far only proteome-derived data are available for this species. Nevertheless, to understand the complex venom evolution in parasitoids, an extended taxon sampling is essential combined with comparative, in-depth venomics studies.

## 2. Results

In this study, we used a proteo-transcriptomic approach to characterize the venom and the possible function of its components from *P. turionellae,* of which thus far only a few, older proteome based studies are available [45]. For characterizing the venom compositions in more detail, including expression levels of the venom components, a combination of proteomics and transcriptomics was needed. Our proteome analysis of the crude venom was combined with RNA Seq data from body tissue and venom gland system transcriptomes. In newly developed analysis pipelines and workflows, including multiple assemblies, secreted proteins in the proteome were matched with gland specific transcripts considering the higher expression levels as important thresholds as well. Several of the identified transcripts that survived our strict proteo-transcriptomic approach match were already known and described venom components of endoparasitoid venoms, such as laccase and phenoloxidase, which were mostly linked to the encapsulation or melanization processes, and several proteinase inhibitors, metalloproteinase M12B, carboxylesterase, and peptidase S1 variants. Most importantly, we identified the possibly paralyzing factor in the venom, an ICK-fold knottin peptide that we named pimplin2. ICKs are well known for their neurotoxicity in venoms from spiders and several other arthropods. Interestingly, our results did not support earlier findings of venom components of *Pimpla* that were identified via proteome-only approaches, such as apamin, melittin and pimplin. The latter has been described as a major paralyzing factor from *P. hypochondriaca*.

### 2.1. Proteo-Transcriptomic as a Strategy to Identify Coding Transcripts Based on Proteomics

All reads of the three generated cDNA libraries from the tissue of the female venom gland system (Vg), female body (BtF), and male body (BtM) were assembled together utilizing an in-house pipeline applying multiple assemblers (Trinity, rnaSPADES with and without error correction), see material and methods. After quality trimming, identical reads were merged, resulting in a total of 448,782 contigs. From those, 391,271 coding regions were identified by the Transdecoder and finally used in all downstream analyses. For each transcript, protein-coding regions were further annotated via Interproscan, BLASTX (e-value ≤ 10^−6^) against the non-redundant NCBI database, ToxProt, and 118 own hmmer profiles (min. bitscore 15) of known venom peptides and proteins. Additionally, JACKhmmer searches (min. bitscore 15) were performed for 151 known single venom protein sequences that were mined manually for parasitoid wasps (see material and methods for details).

**Figure 2:**
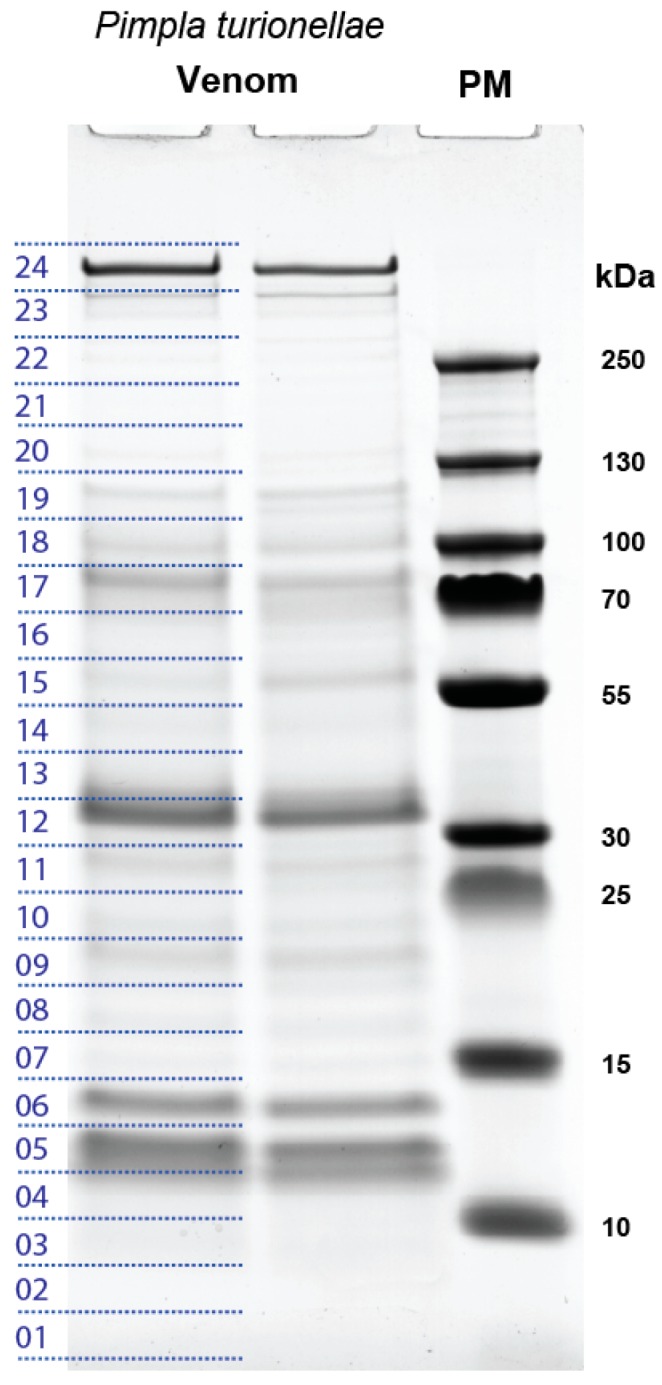
The SDS-PAGE analysis of *P. turionellae* venom proteins. Proteins obtained from the lumen of *P. turionellae* venom glands were separated by SDS-PAGE and stained with Coomassie Brilliant Blue R250. PM = protein marker; numbers on the left indicate the 24 bands cut out from the gel and processed as individual samples for LC-MS/MS. Molecular mass is in kDa.

The components of the crude venom were separated by SDS-PAGE, and the bands were observed from less than 15 kDa to more than 250 kDa (apparent molecular mass), which were separated into 24 samples, see Figure 2. The in-gel digested and trypsinized samples were analyzed via LC-MS/MS. After the fragment identification, the generated transcripts were used as a specific database to match the fragments from the MS analyses with transcripts of secreted proteins using the MASCOT software suite (see material and methods for further details). Subsequently, we only discuss protein families with transcripts for which reads map back that derive from the venom gland tissue library and that were as well identified by at least one transcript via the proteome data (Mascot value ≥ 30 and number of fragments ≥ 2, search window of 0.002 Da, see Table S2). Applying this strict and extensive filtering we avoid the possible over- or mis-interpretation of the transcriptome data as recently discussed [46,47,48].

### 2.2. General Overview of the Transcripts that are Supported by Proteomics

In total, the 339 transcripts that remained after all filtering steps, could generally be separated into three major groups. Group 1: Non-venom related transcripts that were annotated with clear cellular functions such as ribosomal and membrane proteins (175 transcripts), which were not further analyzed. Group 2: Transcripts with annotation similar to known venom protein classes (117 transcripts). Group 3: Transcripts with no similarity to known protein groups (18 transcripts), or with annotations at the amino acid level without conclusive information on protein domain or family (29 transcripts), see Tables S3 and S4. Groups 2 and 3 were further analyzed applying additional thresholds at the expression level to avoid misinterpretation of our data based on the hypotheses that venom proteins should be more highly expressed in the venom gland compared to non-venom-system related body tissue. Our downstream analysis here was thus focused on venom-related candidates that matched known venom proteins classes (group 2), and putative novel venom proteins with inconclusive or no annotation (group 3) that were higher expressed with a TPM (transcripts per million) value of >1 and a two-times higher expression (log2 fold change under minus one) compared to the expression levels of similar or identical transcripts in the female and male body tissue, respectively (see Tables S3–S4).

### 2.3. Composition and Expression of Genes from Known Venom Protein Classes

**Figure 3:**
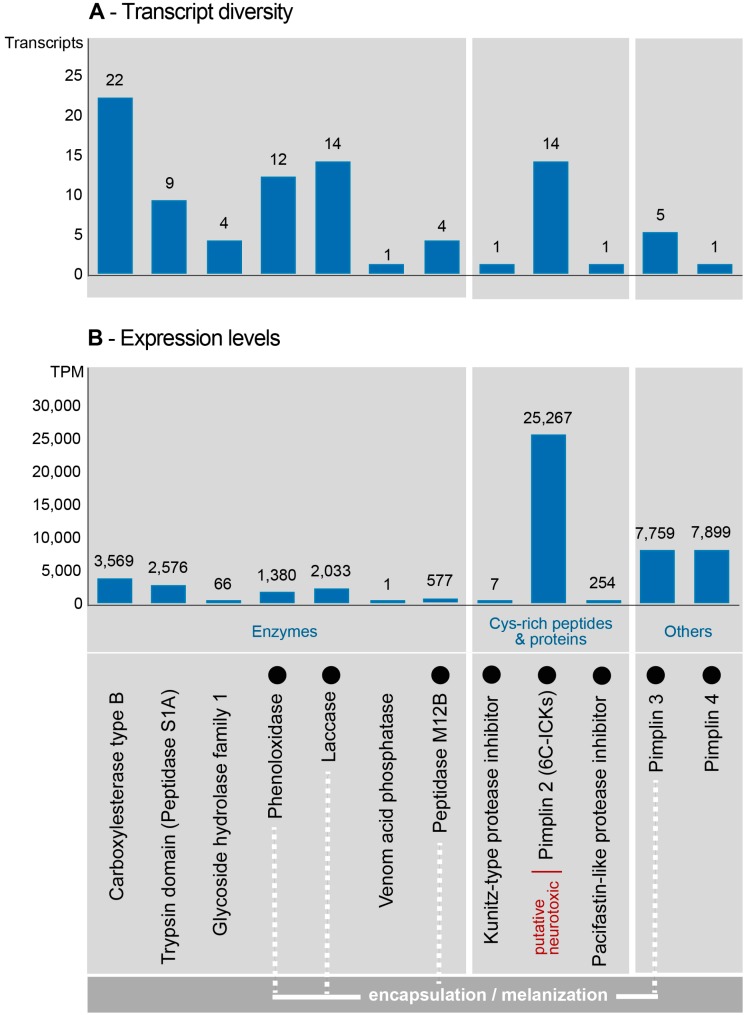
Transcript diversity and expression levels of identified known venom protein families from *P. turionellae* venom glands. The number of transcripts (**A**) and the summarized expression levels in transcript per million (TPM) per protein class (**B**) are shown for functional groups of protein families. The black dots highlight protein families for which sequences from *P. hypochondriaca* were described. Proteins that probably act on the encapsulation and melanization process are highlighted by the white dotted lines. All sequence alignments of known venom proteins are provided in the additional data file 1.

From the final 117 venom function related transcripts, 88 remained that passed our thresholds, and which belonged to 12 known venom protein families (all sequences and alignments are available in the additional data file 1). The identified protein families could be classified into three major groups: Enzymes, cysteine-rich peptides/proteins, and others (Figure 3). Among the identified enzymes in the *P. turionellae* venom, carboxylesterase constitutes the most diverse protein family with 22 transcripts, followed by laccase (14), phenoloxidase (12), S1A superfamily trypsin domain (9), glycoside hydrolase family 1 (4), metallopeptidase M12B (4) and venom acid phosphatase (1) (Figure 3A). 

Identified cysteine-rich venom proteins were new variants of ICK-fold knottin peptides with a 6-C scaffold, which we named Pimplin2 (see Table 1) and kunitz-type and pacifastin-like protease inhibitors. Other components were the here renamed families pimplin3 (including venom protein1, Vpr1) and pimplin4 (including small venom protein2, svp2) that have been described in *P. hypochondriaca* (see Table 1). Most dominantly expressed in the venom were interestingly novel candidates of these three classes: Pimplin2 (TPM 25,267), pimplin4 (TPM 7899), and pimplin3 (TPM 7759). All remaining components show lower levels of expression (see Figure 3B and Table S3).

### 2.4. Novel, Uncharacterized Venom Peptides and Proteins

We also identified 12 putative novel venom proteins. However, for most of those, our search against known venom proteins (hmmer-profiles, ToxProt) and the annotation via InterProscan, remained inconclusive, see Table 2. Manual BLAST search via NCBI revealed many low-scoring matches against bacteria.

## 3. Discussion

Several studies on parasite-host interaction and envenomation effects describe that the idiobiont parasitoid, *P. turionellae*, inflicts the venom injection before oviposition—a quick, obviously permanent paralytic effect on the host followed by the suppression and alteration of the host’s physiology [49,50,51]. The paralyzing component probably prevents, firstly, rapid action from the host to changing its habitat [24]. However, permanent paralysis of the host is reported for only a few endoparasitoids, since most species in this group adopted the koinobiont lifestyle that allows normal development of the host [17]. In contrast, ectoparasitoids generally induce paralysis of the host to prevent any harm to the offspring on/next to it [17]. Other venom components from *P. turionellae* modulate the response of the immune system, such as encapsulation after eggs are placed into the paralyzed host and possibly subsequent melanization of the encapsulated parasitoid. Using a proteo-transcriptomic approach, here we provide a more detailed picture of the two-fold envenomation process (paralysis and suppression of immune response) in *P. turionellae.* The diversity and putative biology of its venom components are discussed in comparison to the few, more in-depth venomics studies of endoparasitoid wasps.

### 3.1. Missing Evidence of the Paralytic Venom Component Pimplin Described for P. hypochondriaca

Until today, only three potentially paralytic or neurotoxic venom components identified in parasitoid wasps were tested for their activity (see Supplementary Table S1). Two of them are *BrhI* and *BrhV* (74 kDa), which were described for the ectoparasitoid wasp *B. hebetor* (Braconidae), and both of which showed potent effects when injected into caterpillars [52]. From the venom of the endoparasitoid *P. hypochondriaca,* a heterodimeric protein (22 kDa) consisting of two polypeptide chains (10.5) and (6.3 kDa) that are linked through a disulfide bond, was isolated and named pimplin. The paralytic effects of pimplin have been shown using adult stages of the housefly *Musca domestica*, the cockroach *Blatella germanica,* and the tomato moth *Lacanobia oleracea* [34]. 

Surprisingly, we found no evidence of this protein in our proteo-transcriptomic data. We can only speculate why pimplin might be missing in the venom of *P. turionellae*. One obvious possibility is that pimplin resembles a species-lineage specific toxin in *P. hypochondriaca* and is thus linked to unique host adaptations. However, given that the two species are not that distinct from each other, the recruitment of this toxin in *P. hypochondriaca* must have occurred very recently. Other reasons could be a false negative hit in our analysis or a possible false-positive identification in the venom of *P. hypochondriaca*.

### 3.2. Pimplin2 (a New ICK Family) Might Act as Paralytic Factor in P. turionellae

**Figure 4:**
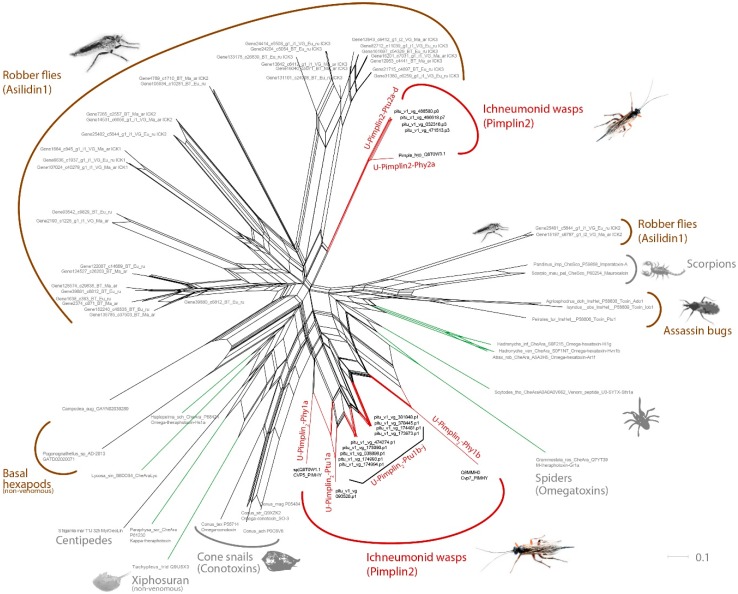
Diversity of known and described cysteine inhibitor knot (ICK) peptides similar to pimplin2. The neighbor-joining network reconstructed in Splitstree 4 [58] is based on protein distances that were optimized using the WAG-Gamma protein substitution model provided in Splitstree [58] and includes known sequences that share the cysteine scaffold of the identified pimplin2 ICK peptide. Known variants from other insect groups such as robber flies or assassin bugs are highlighted in brown. Pimplin2 transcripts from *Pimpla* are colored in red, the spider ICK variants (omegatoxins) are highlighted in green.

The most expressed venom component in *P. turionellae* is the new peptide family pimplin2 that features a structural cysteine inhibitor knot (ICK) motif. Generally, ICKs with varying cysteine scaffolds are widely employed in animal venoms [53], and are of particular applied interest because of their various effects on ion channels. Pimplin2 shows high similarity to known ICK-like toxins explicitly known in spiders, cone snails, assassin bugs, scorpions, and robber flies [53,54]. Interestingly, cysteine-rich venom proteins named cvp1-cvp7 (Cvp = cysteine rich venom protein) have been described already in older studies for *P. hypochondriaca* [42]. In phylogenetic analyses based on structural alignment of known ICK with a similar scaffold, it was revealed that cvp5, cvp3, and cvp7 are highly similar to the new pimplin2 sequences, see Figure 4. Consequentially, the three peptides from *P. hypochondriaca* were renamed to U-Pimplin_2_-Phy2a (Cvp3), U-Pimplin_2_-Phy1b (Cvp5), and U-pimplin_2_-Ph1a (Cvp7), according to the naming scheme in reference [55]. Figure 4 illustrates that two distinct clades of pimplin2 are identified. U-Pimplin_2_-Phy2a and several pimplin2 transcripts of *P. turionellae* are grouping in a clade that is more closely related to asilidin1, an ICK family for which paralytic activity was revealed in robber flies (Asilidae, Diptera). For a more detailed discussion on the evolution of pimplin2 a larger taxon sampling of ichneumonoid wasps would be necessary. Nevertheless, our hypothesis is that functionally, pimplin2 reflects the paralytic component in the venom of *P. turionellae* and possibly in *P. hypochondriaca* as well, which is in congruence with the findings by Parkinson and colleagues [42]. Interestingly, no further components which could potentially have paralytic activity, similar to known parasitoid venom proteins such as arginine kinase from *B. hebetor* and *Aenasius arizonensis* [56,57], were identified in our data.

### 3.3. Venom Components Linked to Encapsulation

Diverse proteins that were shown or were suspected to suppress the encapsulation cascade have been previously identified in the venoms of parasitoid wasps. The protein arsenal acting on this part of the host’s cellular immune response includes several protein classes. Metalloproteases were found in *M. mediator* [15] *E. pennicornis* [59], *P. hypochondriaca* [39], *Toxoneuron nigriceps* [18], *Cotesia chilonis* [29], *A. arizonensis* [57], *N. vitripennis* [16], and *M. mediator* venom (VRF1) [15]. Calretriculin is described for *B. hebetor* [56], *A. arizonensis* [57], *C. rubecula* [60], and *P. puparum* [30]. Venom of *P. hypochondriaca* was shown to contain venom protein 1 (Vpr1) and venom protein 3 (Vpr3) [35,36,43], while Vn.11 was identified in *P. puparum* [61] and the virulence protein P4 (RhoGAP protein) in *L. boulardi* [62,63].

Metallopeptidase M12B (ADAM/reprolysin) has a low expression in *P. turionellae* venom. Reprolysin-type metallopeptidases require zinc for catalysis, and the catalytic site is characterized by a consensus HEXXHXXGXXH sequence [64]. Metallopeptidases act as a general toxic component and show a broad range of activities, including the utilization of host proteins for nutrition, the suppression of host cellular defense, and the degradation of host defense molecules [65,66]. It was shown that a metalloprotease homolog VRF1 from the endoparasitoid wasp, *M. mediator* could modulate egg encapsulation in its host, *Helicoverpa armigera*, by suppressing the Toll pathway [15]. Thus, it can be speculated that M12B peptidase present in *P. turionellae* might as well attack host hemocytes and inhibit encapsulation. This assumption is supported by older in vivo tests that revealed encapsulation inhibitory effects of *P. hypochondriaca* crude venom [39].

Serine proteinases from the peptidase S1A family with trypsin domain that occurs in the venom of *P. turionellae* show a high expression level. Peptidase S1A proteins typically exhibit a conserved catalytic triad of Asp, His, and Ser residues [67] and are known as a widely distributed venom component in hymenopterans, such as woodwasps (*Sirex noctilio*) [68], parasitoid wasps (*C. chilonis* [29]; *A. arizonensis* [57]; *N. vitripennis* [16], *P. puparum* [30]; *Chelonus inanitus* [31], and higher aculeates (*Bombus ignites* [69]; *Vespa magnifica* [70]; *Polybia occidentalis* [71]). Several studies suggest that venoms of parasitoid wasps show in vitro and in vivo cytotoxic activity in host and insect derived cell lines and probably induce apoptosis [37,51,72,73,74,75]. This indicates that the peptidase S1A variants in *P. turionellae* might help to arrest host development and suppress host cellular immune reactions by involving apoptotic processes.

Finally, variants of the venom protein 1 (Vpr1) described in earlier studies of *P. hypochondriaca* venom [35,36,43] likely play a predominant role as possible hemocyte anti-aggregation factor in *P. turionellae*, since the injection of recombinant rVPr1 suppressed the ability of *L. oleracea* and *Mamestra brassicae* to mount hemocyte-mediated immune responses. Protein sequences highly similar to Vpr1 resemble the third most expressed venom component in *P. turionellae,* and we subsequently renamed this protein family pimplin*3.* Interestingly, the recombinant pimplin3 sequence from *P. hypochondriaca* increases, if injected, the sensivity of *M. brassicae* larvae to the commercially available, fungal bio-control agent, *Beauveria bassiana* [44,76,77]. Therefore, the newly described pimplin3 proteins might play an important role as bioactive agent that suppress key immune responses in target pests and increase the efficacy and decrease the use of agrochemicals [44].

### 3.4. Venom Components Involved in the Modulation of Melanization

Encapsulated pathogenic objects like parasitoid eggs usually experience a second host immune response and are melanized in this humoral defense reaction [78]. The melanin capsule can block absorption of nutrients by parasites and may contribute to their killing by starvation [79]. Phenolic intermediates, which are formed during the synthesis of melanin, probably additionally help to kill invading organisms [80]. However, phenoloxidase proteins (POs) of (endo)parasitoid crude venom are thus far only reported from *P. hypochondriaca* [37]. Sequencing of complementary DNA (cDNA) of fractionated crude venom of *P. hypochondriaca* indicated the presence of POs encoded by three genes (POI, II, and III) that derived by gene duplication [38]. POs are also expressed in *P. turionellae* venom (TPM 1380). Using L-DOPA as a substrate, PO activity has been reported from venom of the ectoparasitoid *N. vitripennis* [81], but PO proteins were not identified via proteomics. Instead, a multicopper oxidase, laccase, was found [16], which can catalyze the oxidation of L-DOPA [82]. Laccase (lac1) was also described in *P. hypochondriaca* venom, and the authors suggested that in *P. hypochondriaca* venom laccase and PO proteins may orchestrate L-DOPA oxidizing activity [41]. Laccase is also represented in *P. turionellae* venom (TPM 2033) and potentially has a dual function in endoparasitoid venom based on its involvement in insect cuticle sclerotization [83]. When suppressing the melanization cascade in the host, parasitoid eggs are defenseless against microbes and POs in *P. turionellae* venom could, therefore, initiate defense reactions in case of egg rupture or attacks by microorganisms. Finally, cytotoxic action of *P. hypochondriaca* [84] *and N. vitripennis* [74] venom was inhibited in insect cells using phenoloxidase inhibitor phenylthiourea to (PTU). This indicates that laccase and POs additionally mediate cell death related to the suppression of the host immune response.

### 3.5. Known and Novel Venom Components with Unknown Function

The negative regulation of the serine protease-mediated melanin synthesis is carried out with different types of serine protease inhibitors (SPIs) such as serpins, kunitz-type, and pacifastin [85]. SPIs have been identified in several parasitoid venoms, including *P. puparum* (PpS1V) [33], *L. boulardi* (LbSPNy) [86], *C. chilonis* [29], *N. vitripennis* [16], *Anisopteromalus calandrae* [87], and *P. hypochondriaca* (Cvp2 and Cvp4) [42]. 

The cysteine-rich protein (Cvp2) was first determined in *P. hypochondriaca* venom, but its function remains unclear. These cysteine-rich kunitz-type serine protease inhibitors feature ~60 amino acids stabilized by three disulfide bridges connecting cysteins 1–6, 2–4, 3–5 [88]. In *P. turionellae*, kunitz-type peptides similar to Cvp2 are higher expressed compared to other components (TPM 2194), but we can only speculate that its function is to interfere with the melanin biosynthesis. Another proteinase inhibitor is the pacifastin-like protein (Cvp4), which was identified in *P. hypochondriaca* venom, containing a triplicated six-cysteine motif [42], while *in N. vitripennis*, this motif is repeated four times [16]. In *P. turionellae*, a pacifastin-like protein is moderately expressed, but shows only two repeats of this motif. In addition to their main function, some kunitz-type and pacifastin peptides are also capable of blocking ion channels, especially the voltage gated potassium channels, which are essential for regulating various physiological processes such as blood coagulation or host defense [88].

Venom acid phosphatases were already identified in other hymenopteran species, including *N. vitripennis* [16], *P. puparum* [30], *A. calandrae* [87], *S. noctilio* [68], *B. hebetor* [56], *Apis mellifera* [89], and *P. hypochondriaca*. However, these proteins might only play a secondary role in the venom of *P. turionellae* since they display rather low gene expression levels compared to the other major components. Although the function of venom acid phosphatases is not known yet, and first tests from *P. hypochondriaca* showed no effects on hemocytes from *L. oceracea* [32,90], therefore, a cytotoxic activity is hypothesized. 

Carboxylesterase type B has been reported in ectoparasitoid venoms, including *B. hebetor* [56] and *N. vitripennis* [16], and shows a high number of transcripts (20) and high levels of expression (TPM 3569) in the venom system of *P. turionellae*. Carboxylesterases (COs) are serine hydrolases that catalyze the hydrolysis of carboxylic esters to their component alcohols and acids and are highly diverse in insects [91,92]. COs play an important role in insect metabolism, such as degrading neurotransmitters (cholinesterase) and metabolizing specific juvenile hormones [93,94,95]. Their function in *P. turionellae* remains unclear, but they could be involved in the developmental processes that are controlled by the parasitoid.

Glycoside hydrolase family 1 (GH1) is one of the most abundant venom components of the parasitoids *Psyttalia lounsburyi* and *Psyttalia concolor* (Hymenoptera, Braconidae), which are important bioagents against the olive fruit fly [96]. GH1 enzymes catalyze hydrolysis of glycosidic bonds between carbohydrates and breakdown polysaccharides into smaller products [97]. They are widely distributed in the animal kingdom and play important roles in carbohydrate metabolism, defense, and detoxification [96,98,99,100,101,102]. For *P. turionellae*, it is speculated that GH1 might release host carbohydrates in order to feed the parasitoid larva. Nevertheless, GH1 reflects in our study a rather low expressed component in *P. turionellae* venom.

The venom component expressed at the second highest level in *P. turionellae* we named pimplin4 (TPM 7899), which is a small venom protein that was already described as svp2 (small venom protein2) in *P. hypochondriaca,* [42]. The function of pimplin4 in *P. turionaellae* remains speculative. Interestingly, it shows no similarity to other known proteins and would represent an interesting candidate for activity tests. We refrain from discussing the function of pimplin4 and the potential novel candidates here in detail, because the annotations are inconclusive, and without any further information, it remains to be tested which function they have.

## 4. Conclusions

Hymenopterans belong to one of the most venomous species-rich groups in the animal kingdom, and a large majority of its species are parasitoid wasps. Despite many articles suggesting that hymenopteran venoms are well understood or most of their effector proteins are known, only a few species have been studied in more detail. This is particularly the case for taxa in closer proximity to humans, such as bees and wasps [17]. Parasitoids, especially endoparasitoid wasps, however became of interest rather late, mostly due to their employment in some cases of paralyzing and immune suppressive venoms. These functions are, in particular, of desire for pharmacological and agricultural applications. In this study we describe new variants of pimplin3-like proteins, which could, for example, make pest species more vulnerable to agrochemicals [44], thus increasing their effectiveness while reducing the applied quantities of these toxic substances. Nevertheless, because only few parasitoids have been analyzed using in-depth venomics studies, this leaves a huge potential for applied research untouched. 

Comparative approaches that include more taxa are crucial to understanding venom evolution in general and for particular groups [3]. Especially in mega-diverse groups such as hymenopterans more species need to be studied. Of particular interest are obviously the occurrence of lineage-specific venom proteins, such as the herein named pimplin3 and 4. Some venom proteins that use a common motif like the ICK pimplin2 might be lineage-specific as well after convergent recruitment, or their evolutionary origin could be shared in *P. turionellae* with robber flies. To finally unravel the origin and to understand the mechanisms how these cysteine-rich and other venom proteins evolve the inclusion of comparative genomic data in venomics studies is of utmost importance as shown for asilidin1 in the robber fly *Dasypogon diadema* [54] and for *N. vitripennis* [103].

## 5. Materials and Methods

### 5.1. Rearing and Dissection of P. turionellae Specimens for Proteomics and Transcriptomics

To obtain sufficient specimens for proteome and transcriptomic work, colonies of *P. turionellae* (Hymenoptera, Ichneumonidae) were reared in the lab according to reference [104], using pupal stages of *Galleria mellonella* (greater wax moth) as hosts. Adult parasitoids were collected after hatching from the host pupae and held in 1 L glass jars without a host and fed on 50% (v/v) honey solution.

**Figure 5:**
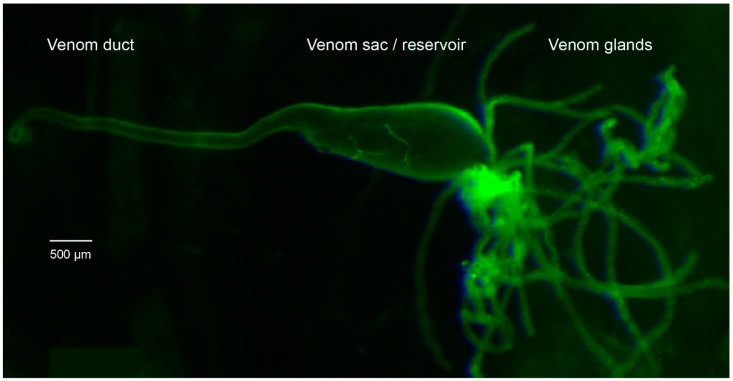
The dissected parts of the venom gland system in *P. turionellae*. All tissue parts (venom duct, venom sac, and venom glands) were together used for the transcriptome and proteome data generation.

For proteomics work, the venom gland system of 25 mated females of *P. turionellae* was dissected after 10–20 days, see Figure 5. All specimens were anesthetized in −80 ˚C for 3 min, and each venom sac was carefully removed from the abdomen on ice in 60 µL sterile PBS buffer and separately transferred into a 0.2 mL microfuge tube with 20 µL of PBS buffer. Each venom sac was then punctured with sterile forceps to obtain the crude venom. All tissue remains were removed, and the crude venom stored at −80 ˚C for subsequent proteome analysis. 

The dissection for venom glands for transcriptomics followed the same protocol, except that 50 venom sacs and ducts had to be used and were finally preserved in 1 mL TRIzol reagent (Invitrogen, Carlsbad, CA, USA). All samples were stored at −80 ˚C. Complementary to the venom gland tissue samples, 3 male and 3 female body tissue samples (excluding the venom apparatus) were prepared and stored separately in TRIzol reagent at −80 ˚C.

### 5.2. RNA Isolation, Library Preparation, and Illumina Sequencing

Total RNA was isolated from pooled female venom glands (Vg), female body tissue (BtF), and male body tissue (BtM) samples using TRIzol according to the manufacturer’s instructions, followed by DNase treatment (Turbo DNase, Thermo Fisher Scientific, Waltham, MA, USA) and further purification using RNA Clean and Concentrator 5 (Zymo Research, Irvine, CA, USA). RNA quantity was determined using an Implen Nanophotometer (Implen Inc., Westlake Vilalge, CA, USA), and the integrity of all RNA samples was verified using an Agilent 2100 Bioanalyzer and an RNA 6000 Nano Kit (Agilent Technologies, Palo Alto, CA, USA). Transcriptome sequencing was carried out on the Illumina HiSeq 3000 platform by GATC Biotech (Konstanz, Germany). Poly-A containing mRNAs were isolated from 1 µg total RNA using oligo-dT attached magnetic beads, the obtained mRNA was fragmented to an average of 250 bp. Afterwards, 150 bp, paired end sequencing libraries were generated using the Illumina TruSeq RNA library preparation kit for each sample. All information and data related to RNASeq was submitted to NCBI, and the raw transcriptome data for Vg (SRR9901353), BtF (SRR9901351), and BtM (SRR9901352) are accessible via the umbrella BioProject PRJNA555750.

### 5.3. Transcriptome Assembly, Read Mapping, and Identification of Venom Proteins

All reads generated by the sequencing provider were processed in an in-house assembly and annotation pipeline of the Animal Venomics group. For maximizing reproducibility, all software components were packed into docker images that can be run with all used settings easily on any Linux system. The input sequence reads were inspected using FastQC (v0.11.7) [105]. Afterwards, all reads were trimmed using Trimmomatic v0.38 [106] (docker image greatfireball/ime_trimmomatic:v0.38) and the following settings: ILLUMINACLIP:/opt/Trimmomatic/adapters/TruSeq3-PE.fa:2:30:10 LEADING:10 TRAILING:10 SLIDINGWINDOW:4:30 MINLEN:120. The resulting trimmed reads were used as input for the assembly based on multiple assemblers, currently Trinity 2.8.4 [107,108] (docker image greatfireball/ime_trinity:v2.6.6_1), and rnaSPAdes v3.12 [109] (docker image greatfireball/ime_spades:3.12.0) with and without error correction. Contigs from all assemblers were subsequently combined to establish a comprehensive assembly in which transcripts were merged that were derived from the different assembler runs and had the same length and 100% identity. The expression levels were quantified for all transcripts by re-mapping the reads to the assemblies using the mapper Hisat2 v2.1.0 [110] (docker image greatfireball/ime_hisat2:v2.1.0) and the quantification tool stringtie v1.3.5 [111,112] (docker image greatfireball/ime_stringtie:v1.3.5). Conversions between SAM and BAM files were performed by samtools v1.3.1 [113].

Open reading frames in transcripts were predicted with Transdecoder v5.0.2 [108] or https://transdecoder.github.io/; docker image greatfireball/ime_transdecoder:5.0.2) and annotated on the amino acid level performing Interproscan v5.27.66 [114] (docker image greatfireball/ime_interproscan:v5.27-66) and BLASTX searches (e-value ≤ 10^−6^) against the NCBI non-redundant and ToxProt databases, see Table S3. Additionally, an in-house database of 118 HMMER profiles of known venom components was utilized to distinguish and annotate possible toxins, venom proteins, and peptides in the transcriptome data (min. bitscore = 15), the used pipeline is accessible via github: (https://github.com/reumont/ av_hmm_pipeline.git). Known sequences of 151 venom components from parasitoid hymenopterans were mined from the literature and NCBI GeneBank to search these single peptides and proteins via JackHMMER in the data (min. bitscore = 15). To identify the unknown or unannotated venom components, transcripts with higher expression values were included as well. The alignments that were used to perform HMMsearches and the sequences that were the base for JackHMMER searches are available as additional data (additional file 3 and 4) in the public database ZENODO (see additional data), where the assembly and Transdecoder prediction files are also accessible (additional files 5 and 6).

To avoid overestimation of the transcriptome data, we only discuss venom gland based transcripts that were identified and supported by proteome data using the transcriptome as a species-specific database for spectral searches in MASCOT, see 5.5 and, Table S2. Additionally, an expression value of >1 TPM (transcripts per kilobase million) was used to reduce false positives transcripts and a two-fold higher expression level cut-off in venom gland samples compared to body tissue samples based on normalized expression values (see Table S3).

### 5.4. SDS-PAGE and LC-MS/MS Analysis of P. turionellae Venom Proteins

Venom proteins were separated by sodium dodecylsulfate polyacrylamide gel electrophoresis (SDS-PAGE) on 4–12% Criterion™ XT gradient gels (BioRad, Feldkirchen, Germany) with XT MES running buffer, see Figure 2. Before loading, samples were mixed with XT sample buffer and reducing agent and heated for 5 min at 95 ˚C. Gels were run for 70 min at 130 V and stained using Coomassie Brilliant blue R250 (Imperial Protein stain, Thermo Scientific). Molecular weights (kDa) of separated venom proteins were assessed using a pre-stained protein marker.

Two lanes of the SDS-PAGE gel were excised into 24 molecular weight fractions each, containing nearly equal staining densities across both lanes (Figure 2). Tryptic digestion was carried out as described by reference [115]. For LC-MS analysis, samples were reconstructed in 50 μL aqueous 1% formic acid, and 1 µL of the peptide mixture was injected onto an UPLC M-class system (Waters, Eschborn, Germany) online coupled to a Synapt G2-si mass spectrometer equipped with a T-WAVE-IMS device (Waters, Eschborn, Germany). Samples were first on-line pre-concentrated and desalted using a UPLC M-Class Symmetry C18 trap column (100 Å, 180 µm x 20 mm, 5 µm particle size) at a flow rate of 15 µL min^−1^ (0.1% aqueous formic acid). Peptides were eluted onto an ACQUITY UPLC HSS T3 analytical column (100 Å, 75 µm X 200, 1.8 µm particle size) at a flow rate of 350 nL/min using an increasing acetonitrile gradient from 2% to 90% B over 65 min (Buffers: A, 0.1% formic acid in water; B, 100% acetonitrile in 0.1% formic acid). The eluted peptides were transferred into the mass spectrometer operated in V-mode with a resolving power of at least 20,000 full width at half height FWHM. All analyses were performed in a positive ESI mode. A 100 fmol/μL human Glu-Fibrinopeptide B in 0.1% formic acid/acetonitrile (1:1 v/v) was infused at a flow rate of 1 μL min^−1^ through the reference sprayer every 45 s to compensate for mass shifts in MS and MS/MS fragmentation mode.

Data were acquired using data-dependent acquisition (DDA). The acquisition cycle for DDA analysis consisted of a survey scan covering the range of m/z 400 to 1800 Da followed by MS/MS fragmentation of the 10 most intense precursor ions collected at 0.5 s intervals in the range of 50 to 2000 m/z. Dynamic exclusion was applied to minimize multiple fragmentations for the same precursor ions. MS data were collected using MassLynx v4.1 software (Waters, Eschborn, Germany). All proteome data files (including the raw data and mzML file) generated in the proteomics analysis are accessible as additional data (additional files 7–10) via the public database ZENODO (https://zenodo.org/record/3545834).

### 5.5. Matching Mass Spectrometry Data with Transcriptome Data

The ion spectra of peptides generated by mass spectrometry were interpreted using MASCOT (v2.6.2, Matrix Science, London, UK) and the generated transcriptome assembly as a specific database. The following searching parameters were applied: Fixed precursor ion mass tolerance of 10 ppm for survey peptide, fragment ion mass tolerance of 0.02 Da, estimated calibration error of 0.002 Da, 1 missed cleavage, fixed carbamidomethylation of cysteines and possible oxidation of methionine. After de-grouping the transcripts identified in MASCOT, only transcripts that matched a minimum MASCOT score of 30 and were identified with a minimum of 2 fragments were finally discussed, see Table S2. All further characterization of transcripts that matched fragments, which were discovered in the MS analysis was conducted based on the MS results in the MASCOT table applying the transcriptome data processing as described in 5.3.

## Figures and Tables

**Table 1:** Renamed known peptides and proteins with novel variants in *P. turionellae*. * Pimplin described in *P. hypochondriaca* was not found in *P. turionellae*, but is listed for completeness. The brackets indicate the domain structure of pimplin 2, the C’s are the pattern of the cysteine scaffold for whole sequences. The lengths range is given for all sequences, including those from *P. hypochondriaca*. All sequence alignments with the named sequence-IDs and corresponding neighbor-joining networks are provided in the additional data file 1.

Name	Structural Fold	Scaffold	Length(aa)	TPM
Pimplin *	Dimeric protein	Prolin scaffold	143	NA
Pimplin2	ICK	X-CX_7_-[C-X_6_-C-X_5–8_-CC-X_2–4_-C-X_6–9_]-X	63–115	25,267
Pimplin3	Protein	Potential P and C scaffold	167–315	7759
Pimplin4	Short protein	No cysteine scaffold, 3 P residues	70–78	7899

**Table 2:** Overview of identified putative novel venom proteins. Shown are the IDs for each transcript that were identified via proteomics, the manual BLAST/annotation results, and the expression levels (TPM). Peptides (<50 aa) and Proteins (>50 aa) are sorted according to their expression levels. Candidates that could be of interest for more detailed analyses, for example, bioactivity tests, are highlighted in light grey. All sequences and alignments of novels are available in the additional data (additional data file 2) deposited in the open access database ZENODO (see additional data).

Name	Transcript ID	Manual BLAST Match	Length(aa)	TPM	Signal Peptide
NovelP1	pitu_v1_174267	Inconclusive-non cytoplasmic domain	126	7746	Yes
NovelP2	pitu_v1_002265	Inconclusive-bacterial	50	6141	No
NovelP3	pitu_v1_377983	Inconclusive-bacterial	17	1209	No
NovelP4	pitu_v1_378290	Inconclusive-bacterial	14	1159	No
NovelP5	pitu_v1_002208	Inconclusive-non cytoplasmic domain	73	288	No
NovelP6	pitu_v1_468063	Inconclusive-non cytoplasmic domain	70	239	Yes
NovelP7	pitu_v1_094627	Inconclusive-non cytoplasmic domain	167	219	Yes
NovelP8	pitu_v1_377800	Inconclusive-non cytoplasmic domain	214	208	Yes
NovelP9	pitu_v1_473891	Inconclusive-bacterial	11	180	No
NovelP10	pitu_v1_176834	Inconclusive-Water bear-uncharacterized	43	102	No
NovelP11	pitu_v1_172572	Inconclusive-bacterial	49	36	No
NovelP12	pitu_v1_285207	Inconclusive-bacterial	19	2	No

## Supplementary Materials

The following are available online at https://www.mdpi.com/2072-6651/11/12/721/s1, Table S1: Gives an overview of studies focused on endoparasitoid venoms. The known and tested biological function of major components and the applied methods to identify the proteins/peptides are illustrated, Table S2: Summarizes the results from the mass spectrometry that were analyzed in Mascot and showed the identified protein families and corresponding expression values, Table S3: Gives an overview of the differently expressed transcripts and the TPM values of reads that map to the venom gland tissue transcriptome. Additionally, the annotation results blasting against the Toxprot, and NCBI databases are given, Table S4: Shows the detailed annotation for each transcript that maps to the venom gland tissue transcriptome based on a scan against the InterPro database. Additional Data: Additional data is provided in the open access database ZENODO (https://zenodo.org/record/3545834) including following additional data files: Additional data file 1: Alignments of known venom proteins, Additional data file 2: Alignments of novel venom proteins, Additional data file 3: Sequence alignments to train HMMsearch, Additional data file 4: Sequences to train JACHHMMERSearch, Additional data file 5: RNASeq *de novo* assembly, Additional data file 6: Coding regions predicted with Transdecoder, Additional data file 7: MassSpec mgf file, Additional data file 8: MassSpec mzML file, Additional data file 9: MassSpec pkl file, Additional data file 10: MassSpec raw data.
